# Association between lipoprotein combine index and all-cause and cardiovascular mortality in patients undergoing peritoneal dialysis: a multicenter retrospective cohort study

**DOI:** 10.3389/fnut.2026.1768195

**Published:** 2026-03-03

**Authors:** Caixia Yan, Qing Zhan, Qingdong Xu, Fenfen Peng, Yueqiang Wen, Na Tian, Xiaoyang Wang, Xiaoran Feng, Xianfeng Wu, Juan Wu, Ning Su, Xingming Tang, Qian Zhou, Jianlong Lu, Yanbing Chen, Xiaojiang Zhan

**Affiliations:** 1 Department of Nephrology, The First Affiliated Hospital, Jiangxi Medical College, Nanchang University, Nanchang, China; 2 Key Laboratory of Urinary System Diseases of Jiangxi Province, Nanchang, China; 3 Department of Endocrinology, The First Affiliated Hospital, Jiangxi Medical College, Nanchang University, Nanchang, China; 4 Department of Nephrology, Jiangmen Central Hospital, Jiangmen, China; 5 Department of Nephrology, Zhujiang Hospital, Southern Medical University, Guangzhou, China; 6 Department of Nephrology, The Second Affiliated Hospital of Guangzhou Medical University, Guangzhou, China; 7 Department of Nephrology, General Hospital of Ningxia Medical University, Yinchuan, China; 8 Department of Nephrology, The First Affiliated Hospital of Zhengzhou University, Zhengzhou, China; 9 Department of Nephrology, Jiujiang No. 1 People’s Hospital, Jiujiang, China; 10 Department of Nephrology, Affiliated Sixth People’s Hospital, Shanghai Jiao Tong University, Shanghai, China; 11 Department of Nephrology, Zhejiang Provincial People's Hospital (Affiliated People's Hospital), Urology and Nephrology Center, Hangzhou Medical College, Hangzhou, Zhejiang, China; 12 Department of Hematology, The Sixth Affiliated Hospital of Sun Yat-sen University, Guangzhou, China; 13 Department of Nephrology, DongGuan SongShan Lake Tungwah Hospital, Dongguan, China; 14 Clinical Trials Unit, Department of Medical Statistics, The First Affiliated Hospital, Sun Yat-sen University, Guangzhou, China

**Keywords:** cardiovascular disease, dyslipidemia, lipoprotein combine index, mortality, peritoneal dialysis

## Abstract

**Background:**

Cardiovascular disease (CVD) is the leading cause of death in patients undergoing peritoneal dialysis (PD). The lipoprotein combine index (LCI), integrating total cholesterol, triglycerides, LDL-C and HDL-C, may better reflect atherogenic burden than traditional single-lipid measures. We hypothesized that higher baseline LCI would be independently associated with increased risks of all-cause and cardiovascular mortality in incident PD patients.

**Methods:**

In this multicenter retrospective cohort, 1,986 incident PD patients from six centers (2005–2021) were analyzed. LCI was divided into quartiles (Q1-Q4). Outcomes were all-cause and CVD mortality. Missing covariates were imputed. Centre-stratified Cox models estimated hazard ratios (HRs), and restricted cubic splines assessed non-linear trends.

**Results:**

Over a median 35-month follow-up, 662 deaths occurred, including 328 CVD deaths. Higher LCI quartiles showed progressively higher mortality. For all-cause death, adjusted HRs (95% CI) were 1.41 (1.10–1.80), 1.59 (1.25–2.02) and 1.70 (1.34–2.15) for Q2-Q4 vs. Q1. For CVD death, HRs were 1.45 (1.03–2.02), 1.29 (0.92–1.82) and 1.68 (1.22–2.33). A non-linear pattern was observed for all-cause mortality, with risk increasing when LCI exceeded ~20. The association with CVD mortality was stronger in younger patients (<60 years) (P interaction = 0.048).

**Conclusion:**

Higher baseline LCI independently predicted all-cause and CVD mortality in PD patients, supporting its usefulness for risk stratification and age-specific lipid management.

## 1 Introduction

Cardiovascular disease (CVD) is the leading cause of death in patients on peritoneal dialysis (PD), contributing to a significant proportion of all-cause mortality within this high-risk group (1). In addition to traditional atherosclerotic risk factors, PD patients face unique metabolic challenges, such as chronic glucose absorption from dialysate, persistent low-grade inflammation, reduced residual renal function (RRF), and protein-energy wasting. These factors collectively drive accelerated atherogenesis and vascular damage (2–5). Consequently, there is an urgent need for improved biomarkers that capture the integrated lipid burden, rather than isolated components to enhance risk stratification in the PD population.

For the general population, traditional measures like total cholesterol (TC), triglycerides (TG), low-density lipoprotein cholesterol (LDL-C), and high-density lipoprotein cholesterol (HDL-C) have long been the standard for cardiovascular risk assessment (6, 7). However, the prognostic utility of these individual lipid indices is often inconsistent and frequently paradoxical in end-stage renal disease (ESRD), especially within PD cohorts (8–10). For example, lower TC or LDL-C concentrations at the start of PD have been paradoxically linked to increased all-cause mortality, a finding often explained by the malnutrition-inflammation complex (8, 9). These observations highlight the inherent limitation of relying on single lipid measurements to accurately assess cardiovascular risk in this specific patient population.

More recently, composite lipid indices, which integrate multiple lipid fractions, have emerged as potentially superior predictors for both cardiovascular and all-cause mortality. One such metric is the Lipoprotein Combine Index (LCI), calculated as (TC × TG × LDL-C) ÷ HDL-C. This novel index encapsulates the overall atherogenic load while factoring in the counter-regulatory influence of HDL-C (11). Studies in non-dialysis populations indicate that elevated LCI levels are significantly associated with coronary artery disease, carotid atherosclerosis, and adverse cardiovascular outcomes. Its predictive capability often matches or surpasses simpler ratios like TC/HDL-C or LDL-C/HDL-C (12, 13). Despite these encouraging preliminary data, the clinical relevance of the LCI in the dialysis setting remains unexplored.

Patients on PD typically display a distinct lipid profile: elevated TG, reduced HDL-C, variable LDL-C levels, and an altered lipoprotein particle composition (10, 14). These alterations stem from metabolic challenges specific to dialysis, notably peritoneal glucose load, insulin resistance, and dysregulated hepatic lipid metabolism (2, 10). Moreover, systemic inflammation, oxidative stress, and the progressive loss of RRF further compromise lipoprotein clearance, potentially making conventional lipid metrics less reliable for outcome prediction (3, 4). Considering this complex pathophysiology, it is plausible that the LCI could function as a more sensitive, integrated measure of dyslipidemia-related cardiovascular risk in PD patients.

While the LCI has been assessed across various cardiometabolic contexts, its prognostic significance specifically within the PD population is still largely unknown. Therefore, we undertook a multicenter retrospective cohort study to investigate whether baseline LCI is associated with all-cause and cardiovascular mortality among patients newly initiating PD. We hypothesized that an elevated LCI would correlate with increased risks of adverse outcomes, and that our results could provide novel insights for lipid-related risk stratification and future management strategies in this patient group.

## 2 Materials and methods

### 2.1 Study design and population

This multicenter retrospective cohort study was conducted among adult patients who initiated PD between January 2005 and December 2021 in six tertiary PD centers in China. The study followed the STROBE guidelines for observational studies. Eligible participants were those aged ≥ 18 years who commenced PD as their first renal replacement therapy and remained on PD for ≥ 3 months. Exclusion criteria included: (1) prior hemodialysis or kidney transplantation; (2) active malignancy; (3) acute infection at baseline; (4) outliers of lipid data; and (5) missing baseline lipid parameters required for calculating the LCI.

The study protocol adhered to the principles of the Declaration of Helsinki and was approved by the Ethics Committee of the First Affiliated Hospital of Nanchang University [Approval No. 852 IIT (2025)] and by the ethics committees of participating centers. Patient data were anonymized prior to analysis.

### 2.2 Data collection and biochemical measurements

Baseline data were obtained from electronic medical records at the time of PD initiation. Demographic and clinical variables included age, sex, body-mass index (BMI), systolic and diastolic blood pressure (SBP, DBP), primary kidney disease, diabetes mellitus, hypertension, history of CVD, and medication use (statins, aspirin, *β*-blockers, renin–angiotensin-system inhibitors, and calcium-channel blockers). PD-specific parameters, such as Kt/V, weekly CrCl, and peritoneal transport status, were not included, as these are typically measured during follow-up after the initiation of PD. Although we considered incorporating these PD-related data from the first 6 months of dialysis, significant missing data across the multicenter cohort led to their exclusion to preserve the integrity and robustness of the analysis.

Laboratory variables included fasting lipid profile, TC, TG, LDL-C, and HDL-C, together with hemoglobin, serum albumin (ALB), uric acid (UA), alkaline phosphatase (ALP), calcium, phosphate, C-reactive protein (CRP), creatinine, and RRF (estimated by 24-h urine collection). All tests were performed in the certified laboratories of each center using unified methods and regular external quality control. The LCI was computed as (TC × TG × LDL-C)/HDL-C, with all lipid values expressed in mmol/L. Participants were categorized into quartiles (Q1 - Q4) according to baseline LCI values, with Q1 indicating the lowest and Q4 the highest lipid burden. The cut-points for quartiles were determined using the 25th, 50th, and 75th percentiles of LCI values.

### 2.3 Follow-up and outcomes

Patients were reviewed quarterly in each PD center and contacted monthly by trained dialysis nurses via outpatient visits or telephone. The primary endpoint was all-cause mortality; the secondary endpoint was cardiovascular mortality. Cardiovascular death was defined as death resulting from acute myocardial infarction, heart failure, arrhythmia, sudden cardiac death, or cerebrovascular events (stroke or intracranial hemorrhage) (15).

Follow-up continued until death, kidney transplantation, transfer to hemodialysis, loss to follow-up, or 31 December 2021, whichever occurred first. When death occurred outside hospital, the cause was verified by physician review of family-reported circumstances and prior medical records.

### 2.4 Statistical analysis

Continuous variables were presented as mean ± standard deviation (SD) or median (interquartile range, IQR) according to data distribution, and categorical variables as counts (percentages). Baseline differences across LCI quartiles were compared using one-way ANOVA or Kruskal-Wallis tests for continuous data and the chi-square test for categorical variables.

Missing baseline covariates were imputed using multiple imputation by chained equations (MICE) with predictive mean matching (five imputations, fifty iterations, seed = 500). Although some variables, including pre-existing CVD, had higher missing proportions (≈48%), all clinically relevant variables were included in the imputation model to minimize bias and preserve statistical power. The results from the multiple imputed datasets were pooled using Rubin’s rules to obtain unbiased overall estimates and standard errors. To further assess the impact of missing data, we performed a Missing Not at Random (MNAR) sensitivity analysis. We used MICE with the selection model for imputation under the MNAR assumption, generating five imputed datasets. The results from these datasets were pooled using Rubin’s rules. The association between LCI and mortality outcomes was evaluated using Cox proportional hazards regression models, with results stratified by center. We compared the findings from the MNAR analysis with those from the original analysis (based on the MAR assumption) to assess whether the missing data affected the observed associations.

Survival probabilities were estimated by Kaplan–Meier curves, and log-rank tests were applied to assess inter-quartile differences. The association between LCI and mortality was examined using Cox proportional-hazards regression models, reporting hazard ratios (HRs) and 95% confidence intervals (CIs). The proportional-hazards assumption was verified by Schoenfeld residuals. To account for potential baseline hazard differences among participating PD centers, all Cox models were stratified by center (i.e., strata(center)). Three hierarchical models were constructed: Model 0: unadjusted; Model 1: adjusted for age and sex; Model 2: further adjusted for BMI, SBP, DBP, diabetes, pre-existing CVD, hemoglobin, serum ALB, UA, calcium, phosphate, CRP, ALP, RRF, and use of statins or aspirin. In the multivariate Cox proportional hazards regression, variables with a *p* value < 0.1 in univariate analysis were initially selected. Additional variables were included based on clinical relevance and their known association with lipid metabolism and mortality.

To explore non-linear relationships between continuous LCI and mortality, restricted cubic spline (RCS) analyses were performed. Pre-specified subgroup analyses were conducted according to sex (male/female), age (< 60 vs. ≥ 60 years), diabetes (yes/no), CVD (yes/no), serum ALB (< 35 vs. ≥ 35 g/L), and RRF (<median vs. ≥ median), with multiplicative interaction terms tested for significance.

Fine-Gray competing-risk models were used for cardiovascular mortality, treating non-cardiovascular death as a competing event. Sensitivity analyses were undertaken by (1) excluding deaths within 6 months of PD initiation, (2) reclassifying LCI into tertiles instead of quartiles, and (3) repeating the analysis using complete-case data without imputation.

All analyses were performed using R (version 4.3.3). Two-sided *p* values < 0.05 were considered statistically significant.

## 3 Results

### 3.1 Baseline characteristics of the study population

**Figure 1:**
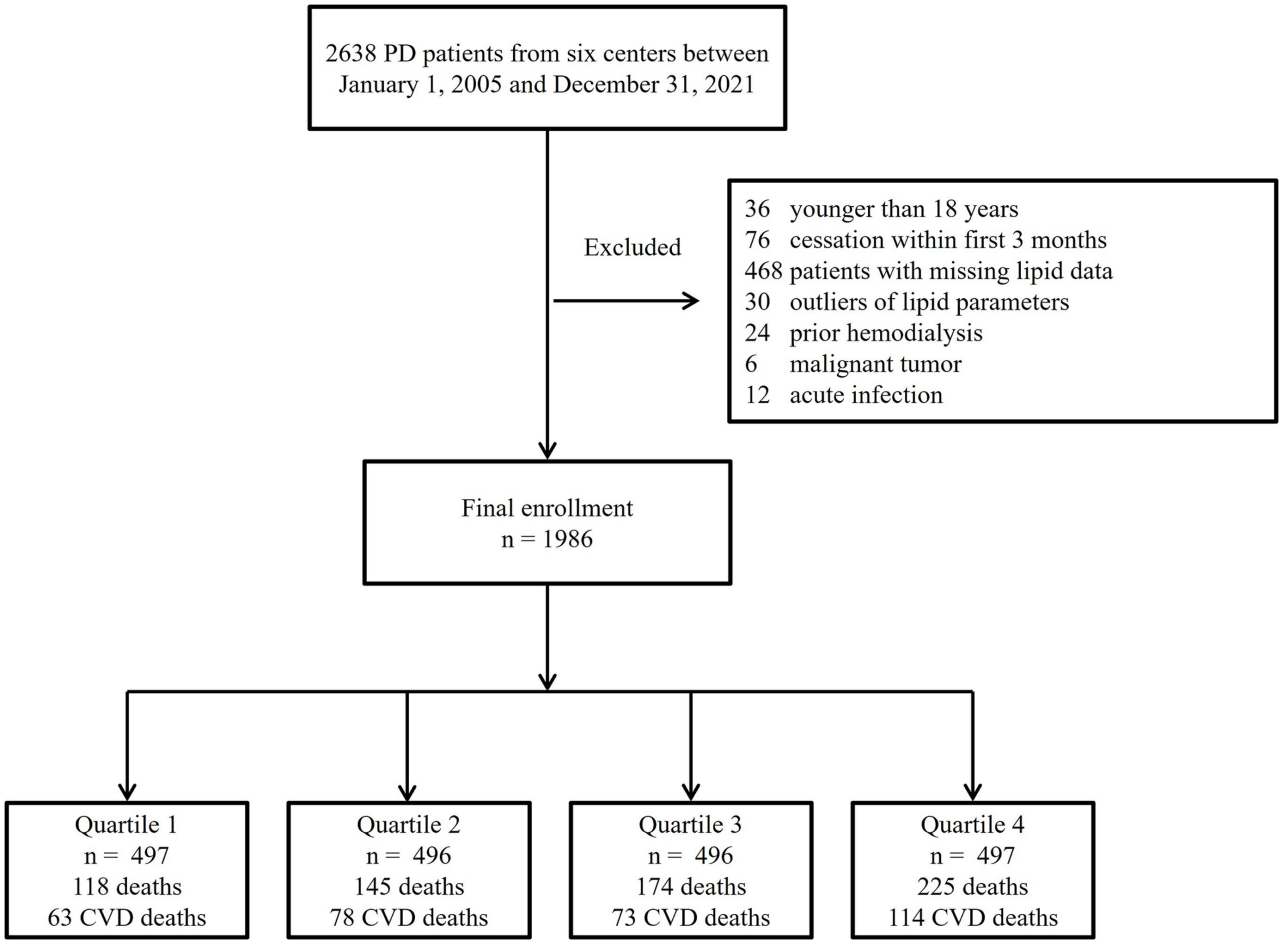
Flowchart of patient selection.

A total of 1,986 incident PD patients were included in the final analysis (Figure 1). The median follow-up duration was 35 months (interquartile range 18–57). During follow-up, 662 (33.3%) patients died, of whom 328 (49.5%) deaths were attributed to cardiovascular causes.

Patients were categorized into LCI quartiles (Q1 ≤ 7.03, Q2 7.03–13.57, Q3 13.57–28.71, Q4 > 28.71). Across increasing quartiles, BMI and the prevalence of diabetes were higher, while HDL-C was lower; TC, TG and LDL-C were progressively higher (all P for trend < 0.001). Hemoglobin and serum ALB were modestly higher in the highest LCI quartile, whereas CRP did not differ materially between groups. SBP and DBP showed no significant differences across quartiles. The proportion of men was lower in Q4 than Q1, statin use was more frequent with higher LCI, and aspirin use was similar across groups. Calcium was slightly higher and phosphate slightly lower with increasing LCI. Although a history of CVD did not differ significantly across quartiles, this finding may partly reflect the substantial missingness for this variable (48.6%), which could limit the reliability of the comparison (Table 1).

**Table 1:** Baseline characteristics of the study population according to Lipoprotein Combine Index (LCI) quartiles.

Variable	Total	Q1 (≤ 7.03)	Q2 (7.03–13.57)	Q3 (13.57–28.71)	Q4 (> 28.71)	*P* (global)	*P* for trend	Missing
	*N* = 1,986	*n* = 497	*n* = 496	*n* = 496	*n* = 497			
Age (years)	51.8 ± 14.5	50.4 ± 14.7	51.1 ± 14.4	51.0 ± 14.4	54.7 ± 14.1	< 0.001	< 0.001	0 (0.0%)
BMI (kg/m^2^)	22.0 ± 3.3	21.3 ± 3.0	22.1 ± 3.1	22.1 ± 3.5	22.6 ± 3.5	< 0.001	< 0.001	0 (0.0%)
SBP (mmHg)	143.6 ± 23.8	143.8 ± 24.8	145.3 ± 23.4	142.8 ± 23.3	142.6 ± 23.8	0.350	0.216	168 (8.5%)
DBP (mmHg)	85.3 ± 14.5	85.5 ± 15.4	86.2 ± 14.2	85.4 ± 14.5	84.1 ± 13.9	0.151	0.094	156 (7.9%)
Hemoglobin (g/L)	92.8 ± 22.9	84.8 ± 20.4	92.3 ± 22.8	95.4 ± 24.2	98.5 ± 21.6	< 0.001	< 0.001	0 (0.0%)
ALB (g/L)	35.6 ± 5.6	35.2 ± 5.1	35.3 ± 5.9	35.8 ± 5.4	36.1 ± 6.1	0.025	0.003	0 (0.0%)
ALP (U/L)	79.2 ± 30.9	81.0 ± 32.5	79.5 ± 30.2	79.6 ± 30.8	76.5 ± 29.9	0.178	0.042	125 (6.3%)
UA (μmol/L)	420.1 ± 110.6	424.4 ± 115.8	416.2 ± 113.2	421.1 ± 109.6	418.6 ± 103.4	0.684	0.569	3 (0.2%)
CHOL (mmol/L)	4.7 ± 1.3	3.6 ± 0.8	4.3 ± 0.9	4.9 ± 1.0	5.9 ± 1.2	< 0.001	< 0.001	0 (0.0%)
TG (mmol/L)	1.6 ± 0.8	0.8 ± 0.3	1.2 ± 0.4	1.7 ± 0.6	2.5 ± 0.7	< 0.001	< 0.001	0 (0.0%)
LDL-C (mmol/L)	2.7 ± 1.0	1.9 ± 0.6	2.5 ± 0.7	2.9 ± 0.9	3.5 ± 1.0	< 0.001	< 0.001	0 (0.0%)
HDL-C (mmol/L)	1.2 ± 0.4	1.3 ± 0.4	1.2 ± 0.4	1.1 ± 0.3	1.0 ± 0.3	< 0.001	< 0.001	0 (0.0%)
LCI	13.6 (7.0, 28.7)	4.3 (2.9, 5.6)	9.9 (8.5, 11.7)	19.5 (16.3, 23.9)	43.8 (35.5, 57.7)	< 0.001	< 0.001	0 (0.0%)
CRP (mg/L)	3.4 (1.3, 10.1)	3.5 (1.4, 10.5)	3.5 (1.2, 10.2)	3.4 (1.4, 9.2)	3.3 (1.2, 10.5)	0.931	0.526	426 (21.5%)
Ca (mmol/L)	2.1 ± 0.3	2.0 ± 0.3	2.1 ± 0.3	2.2 ± 0.3	2.2 ± 0.3	< 0.001	< 0.001	26 (1.3%)
P (mmol/L)	1.7 ± 0.5	1.7 ± 0.5	1.7 ± 0.5	1.7 ± 0.5	1.6 ± 0.5	0.009	0.001	23 (1.2%)
Sex, male (%)	1,082 (54.5)	305 (61.4)	272 (54.8)	254 (51.2)	251 (50.5)	0.002	< 0.001	0 (0.0%)
Diabetes (%)	464 (23.4)	90 (18.1)	114 (23.0)	106 (21.4)	154 (31.0)	< 0.001	< 0.001	1 (0.1%)
History of CVD (%)	593 (29.9)	158 (31.8)	153 (30.9)	138 (27.8)	144 (29.0)	0.568	0.320	965 (48.6%)
Use of aspirin (%)	257 (12.9)	55 (11.1)	62 (12.5)	71 (14.4)	68 (13.8)	0.509	0.200	363 (18.3%)
Use of statins (%)	308 (15.5)	62 (12.6)	69 (14.0)	80 (16.2)	96 (19.3)	0.031	0.003	271 (13.6%)
Centers								
1	857 (43.15)	287 (57.75)	232 (46.77)	206 (41.53)	132 (26.56)			
2	571 (28.75)	83 (16.70)	134 (27.02)	141 (28.43)	213 (42.86)			
3	198 (9.97)	60 (12.07)	51 (10.28)	40 (8.06)	47 (9.46)			
4	182 (9.16)	16 (3.22)	43 (8.67)	60 (12.10)	63 (12.68)			
5	117 (5.89)	26 (5.23)	24 (4.84)	35 (7.06)	32 (6.44)			
6	61 (3.07)	25 (5.03)	12 (2.42)	14 (2.82)	10 (2.01)			

BMI, body mass index; SBP, systolic blood pressure; DBP, diastolic blood pressure; CVD, cardiovascular disease; ALB, albumin; ALP, alkaline phosphatase; Ca, serum calcium; P, serum phosphate; CHOL, total cholesterol; TG, triglyceride; HDL-C, high-density lipoprotein cholesterol; LDL-C, low-density lipoprotein cholesterol; LCI, lipoprotein combine index; CRP, C-reactive protein; UA, uric acid. (1) Data are presented as mean ± SD, median (IQR), or n (%), as appropriate. Units are provided in parentheses. (2) Quartiles were defined using observed LCI values before imputation; cut-points are shown in the column headers. (3) Global p-values (P global) and P for trend were obtained from regression models across multiply imputed datasets and combined using Rubin’s rules: linear models for continuous variables (with transformation if needed) and logistic models for binary variables. (4) “Missing” indicates the number (percentage) of missing observations before imputation.

### 3.2 Association between LCI and mortality

**Figure 2:**
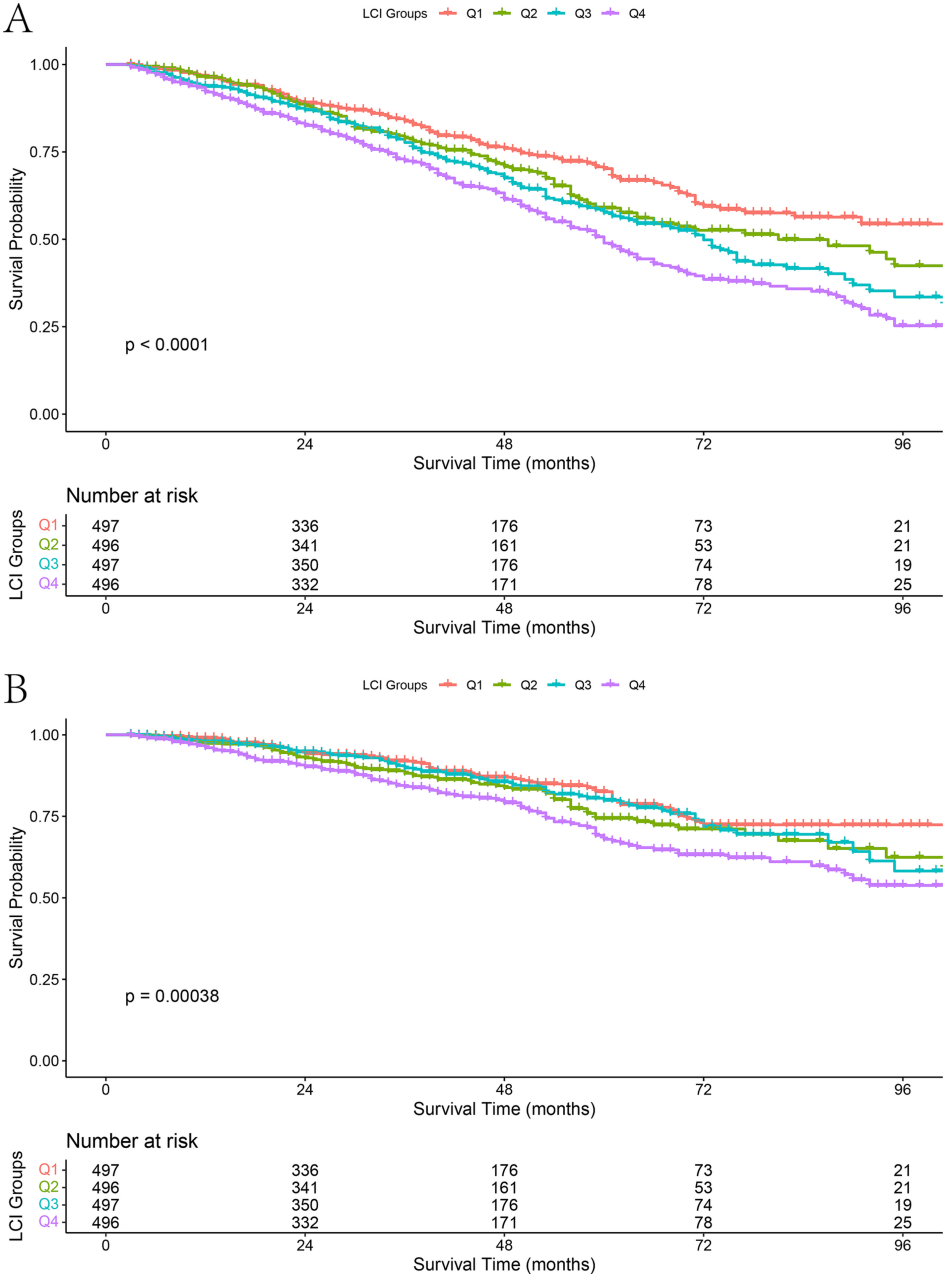
Kaplan–Meier survival curves for all-cause and cardiovascular mortality according to LCI quartiles. **(A)** All-cause mortality; **(B)** Cardiovascular mortality.

During the follow-up, the cumulative survival rates for both all-cause and cardiovascular mortality decreased progressively with increasing LCI quartiles (Figure 2). Kaplan–Meier curves showed significant between-group differences [log-rank *p* < 0.0001 for all-cause mortality (A); *p* = 0.00038 for cardiovascular mortality (B)].

In the multivariable Cox proportional-hazards models (Table 2), higher LCI was independently associated with increased risks of both all-cause and cardiovascular mortality. Using the lowest quartile (Q1) as the reference, the adjusted HRs for all-cause mortality were 1.41 (95% CI 1.10–1.80) for Q2, 1.59 (95% CI 1.25–2.02) for Q3, and 1.70 (95% CI 1.34–2.15) for Q4 (P for trend < 0.001). Similarly, for cardiovascular mortality, the corresponding HRs were 1.45 (1.03–2.02), 1.29 (0.92–1.82), and 1.68 (1.22–2.33) (P for trend = 0.006). When analyzed as a continuous variable, each 1- SD increase in LCI was associated with a 16% higher risk of all-cause mortality (HR 1.16, 95% CI 1.08–1.24; *p* < 0.001) and a 18% higher risk of cardiovascular mortality (HR 1.18, 95% CI 1.07–1.31; *p* = 0.001).

**Table 2:** Association between Lipoprotein Combine Index and mortality outcomes in peritoneal dialysis patients.

Variable	Model 0		Model 1		Model 2	
	HR (95% CI)	*P* value	HR (95% CI)	*P* value	HR (95% CI)	*P* value
All-cause mortality						
LCI Q2 vs. Q1	1.35 (1.06–1.72)	0.017	1.34 (1.05–1.71)	0.019	1.41 (1.10–1.80)	0.007
LCI Q3 vs. Q1	1.56 (1.24–1.97)	< 0.001	1.47 (1.17–1.86)	0.001	1.59 (1.25–2.02)	< 0.001
LCI Q4 vs. Q1	1.99 (1.59–2.49)	< 0.001	1.59 (1.27–1.99)	< 0.001	1.70 (1.34–2.15)	< 0.001
*P* for trend		< 0.001		< 0.001		< 0.001
Continuous LCI (per 1-SD increase)	1.21 (1.14–1.29)	< 0.001	1.15 (1.08–1.24)	< 0.001	1.16 (1.08–1.24)	< 0.001
Cardiovascular mortality						
LCI Q2 vs. Q1	1.36 (0.98–1.90)	0.069	1.38 (0.99–1.92)	0.060	1.45 (1.03–2.02)	0.032
LCI Q3 vs. Q1	1.24 (0.89–1.74)	0.211	1.21 (0.86–1.69)	0.278	1.29 (0.92–1.82)	0.139
LCI Q4 vs. Q1	1.88 (1.38–2.56)	< 0.001	1.59 (1.17–2.18)	0.004	1.68 (1.22–2.33)	0.002
*P* for trend		< 0.001		0.010		0.006
Continuous LCI (per 1-SD increase)	1.22 (1.11–1.33)	< 0.001	1.18 (1.07–1.30)	0.001	1.18 (1.07–1.31)	0.001

These findings remained consistent when competing non-cardiovascular deaths were considered using the Fine-Gray subdistribution hazards model, where the highest quartile had a 54% higher subdistribution hazard for cardiovascular mortality compared with Q1 (sHR 1.54, 95% CI 1.11–2.12, *p* = 0.009) (Supplementary Table S1).

### 3.3 Non-linear and subgroup analyses

**Figure 3:**
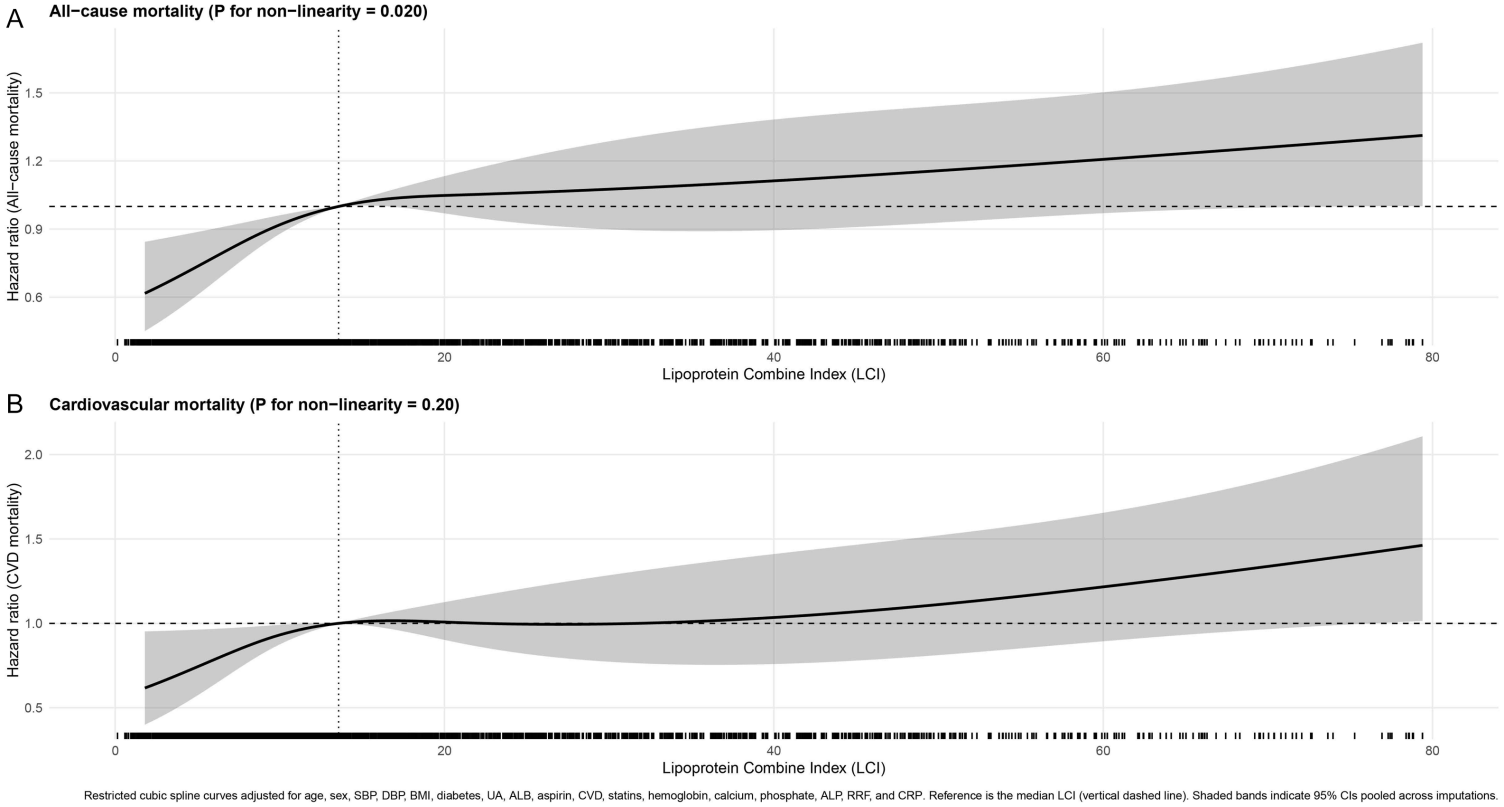
Restricted cubic spline analysis for the association between LCI and mortality. **(A)** All-cause mortality; **(B)** Cardiovascular mortality. The solid lines represent adjusted hazard ratios (HRs), and the shaded areas indicate 95% confidence intervals, with the median LCI as the reference.

RCS analyses demonstrated a non-linear, positive association between LCI and all-cause mortality (P for non-linearity = 0.020), with mortality risk rising sharply when LCI exceeded approximately 20 (Figure 3). In contrast, the association with cardiovascular mortality appeared linear (*p* = 0.20).

**Figure 4:**
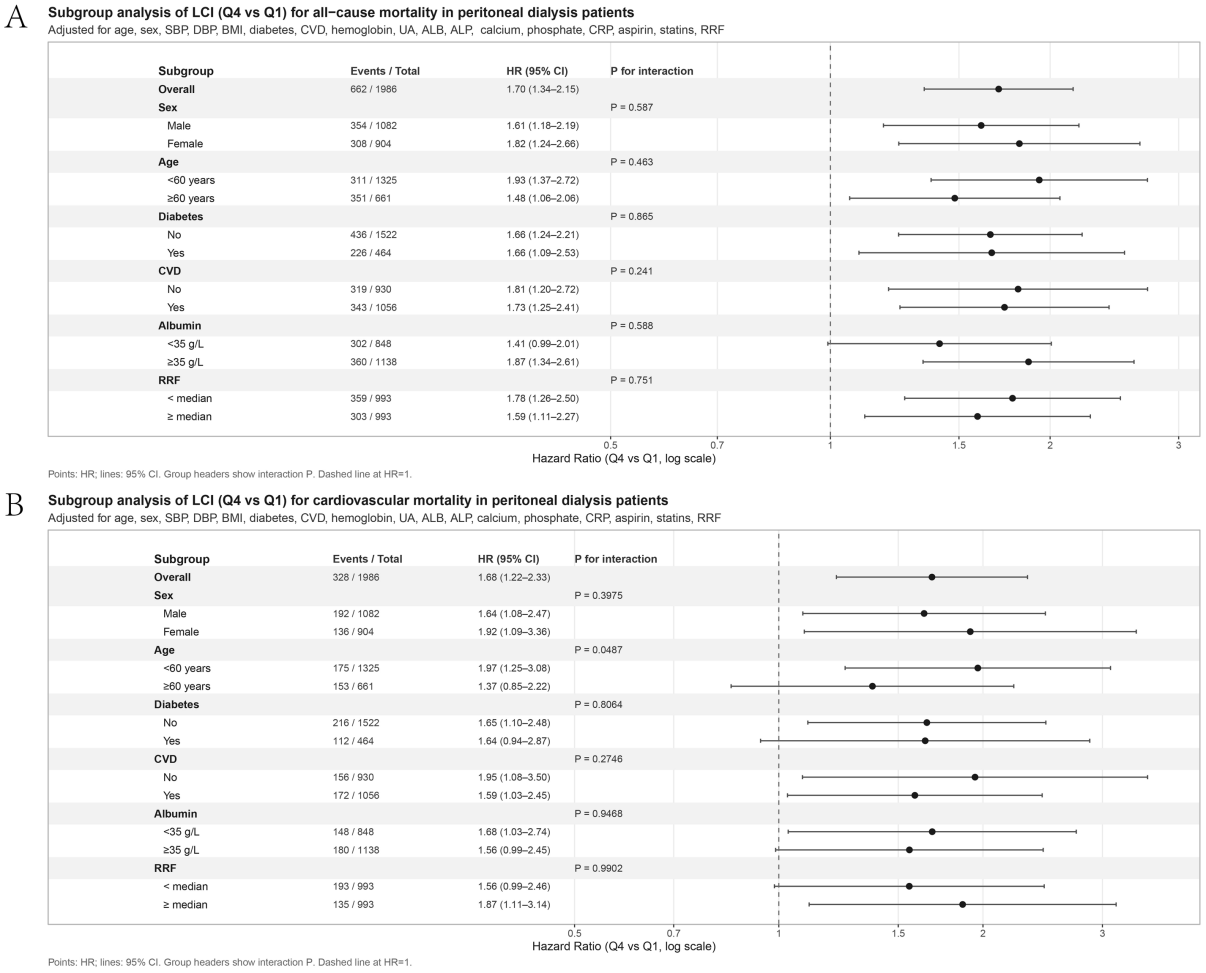
Forest plots of subgroup analyses for the association between LCI and mortality. **(A)** All-cause mortality; **(B)** Cardiovascular mortality. Hazard ratios (HRs) and 95% CIs are shown for LCI Q4 vs. Q1, adjusted for confounders.

Subgroup analyses (Figure 4) showed that the positive association between high LCI and mortality was broadly consistent across sex, age (< 60 vs. ≥ 60 years), diabetes status (yes vs. no), pre-exist CVD (yes vs. no), serum ALB (< 35 vs. ≥ 35 g/L), and RRF categories (< medium vs. ≥ medium) (all P for interaction > 0.05). Notably, for cardiovascular mortality, the overall association persisted, and a significant interaction by age was observed, with a stronger effect among younger patients (< 60 years) (P for interaction = 0.0487).

### 3.4 Sensitivity analyses

Three sensitivity analyses were performed to assess the robustness of the findings. First, after excluding patients who died within the first six months of PD initiation (*n* = 1,892), the associations between LCI and mortality persisted. The adjusted HRs (Model 2) for Q4 versus Q1 were 1.74 (95% CI 1.33–2.29, *p* < 0.001) for all-cause mortality and 1.73 (95% CI 1.20–2.49, *p* = 0.003) for cardiovascular mortality; each 1-SD increase in LCI corresponded to a 17–20% higher risk of death (Supplementary Table S2). Second, when LCI was categorized into tertiles rather than quartiles, the direction and magnitude of the associations remained unchanged. Compared with T1, the adjusted HRs for T3 were 1.55 (95% CI 1.23–1.94) for all-cause mortality and 1.47 (95% CI 1.08–2.01) for cardiovascular mortality (both P for trend < 0.05) (Supplementary Table S3). Third, In the complete-case analysis including 550 patients (27.7% of the total cohort), the associations between LCI and mortality were attenuated and became statistically non-significant (Q4 vs. Q1: HR 1.05 (0.70–1.57) for all-cause and 0.87 (0.52–1.46) for cardiovascular mortality; both *p* > 0.05) (Supplementary Table S4). Given the small sample size and possible selection bias among complete cases, these findings were interpreted as reflecting limited statistical power rather than a genuine absence of association. The MNAR sensitivity analysis (Supplementary Table S5) demonstrated that the relationship between LCI and both all-cause mortality and cardiovascular mortality remained consistent with the results from the original analysis (Table 2). Specifically, the hazard ratios for LCI in the MNAR analysis were similar to those observed under the MAR assumption, suggesting that missing data did not significantly alter the observed associations. These findings support the robustness of our results despite the high rate of missing data.

Overall, the sensitivity and competing-risk analyses (Supplementary Tables S1–S5) generally supported the robustness of the association between higher LCI and increased mortality among PD patients, although the effect estimates in the complete-case analysis were attenuated and no longer statistically significant.

### 3.5 Summary of findings

Higher baseline LCI levels were independently and progressively associated with increased all-cause and cardiovascular mortality in PD patients. The association exhibited a non-linear pattern for all-cause mortality and remained consistent across most subgroups and analytical strategies.

## 4 Discussion

This multicenter cohort provides novel evidence that the LCI is independently associated with both all-cause and cardiovascular mortality among incident PD patients. After comprehensive adjustment for demographic, clinical, and biochemical factors, higher LCI was linked to greater mortality risk, with a non-linear association for all-cause mortality and an approximately linear pattern for cardiovascular death. These findings suggest that LCI reflects an integrated lipid-inflammatory and nutritional burden that is not captured by traditional lipid parameters alone. The significant interaction by age, indicating a stronger association among patients who were younger at PD initiation, suggests that metabolic and inflammatory vulnerability may be more apparent in this subgroup, while in older patients competing risks (e.g., frailty, infection, malnutrition-inflammation) could attenuate the observable relation between LCI and mortality.

In addition to the LCI, other lipid markers, such as non-HDL-C, lipoprotein(a) [Lp(a)], and the TG/HDL-C ratio, are valuable for cardiovascular risk assessment in PD patients. Non-HDL-C reflects the atherogenic burden in PD patients with altered lipid metabolism, and Yu et al. (16) found that elevated non-HDL-C levels are associated with increased cardiovascular mortality. Similarly, Lp(a) has been shown to increase cardiovascular risk, though our previous research found it to be associated with a lower risk of hemorrhagic stroke in PD patients (17). Furthermore, the TG/HDL-C ratio is a reliable predictor of mortality in PD patients, with higher ratios correlating with increased mortality in our previous study (18). Recent studies have also highlighted the importance of including these markers in cardiovascular risk models to better capture residual risk, particularly in patients with well-controlled LDL-C levels (19). These lipid indices, when integrated with the LCI, offer a more comprehensive approach to cardiovascular risk stratification and might guide more personalized management strategies in PD populations.

Several interrelated mechanisms likely explain why an elevated LCI is associated with higher mortality in the PD setting. First, the LCI integrates both pro-atherogenic and anti-atherogenic lipid components into a single score. PD patients frequently develop dyslipidemia marked by hypertriglyceridemia, elevated LDL-C, and reduced HDL-C, driven by factors such as chronic glucose absorption from dialysate, protein loss, and impaired lipoprotein lipase activity (10, 20). This specific lipid combination fosters the formation of small, dense LDL particles and compromises reverse cholesterol transport, accelerating the process of atherosclerosis. Second, a high LCI may serve as an indicator of an underlying systemic pro-inflammatory state. Chronic inflammation and oxidative stress activate cytokines (IL-6, TNF-*α*) and hepatic lipogenesis, leading to further dyslipidemia, while oxidized LDL perpetuates endothelial dysfunction and vascular calcification (21–23). Third, in the PD milieu, peritoneal protein loss contributes to hypoalbuminemia (24), altering lipoprotein composition and function. Albumin depletion impairs lipid binding and increases circulating free fatty acids, augmenting lipid toxicity and mitochondrial dysfunction (25, 26).

The observed non-linear pattern for all-cause mortality may represent a threshold effect: when LCI exceeds approximately 20, compensatory mechanisms, such as antioxidant defenses and residual HDL functionality, become overwhelmed, resulting in a steep rise in risk. Conversely, the linear trend for cardiovascular mortality reflects cumulative exposure to atherogenic lipids driving progressive arterial injury without a distinct inflection point. Finally, the stronger association in younger patients may indicate that the metabolic and vascular insults of elevated LCI exert more prominent effects before competing risks (infection, frailty, or malnutrition) dominate at older ages.

Previous studies on lipid profiles and prognosis in dialysis have yielded inconsistent findings, partly due to the so-called “reverse epidemiology” phenomenon in malnourished or inflamed patients (8, 9). Some reported paradoxical associations where lower TC or LDL-C predicted higher mortality (9, 27). Our results contrast with those findings by demonstrating that when the combined lipid burden is evaluated through LCI, the association with mortality is direct and monotonic. This aligns with recent evidence from maintenance hemodialysis cohorts showing that atherogenic indices such as the atherogenic index of plasma (AIP = log [TG/HDL-C]) and the remnant cholesterol/HDL-C ratio demonstrate prognostic utility for cardiovascular outcomes, often comparable to or better than single lipid markers (28, 29). In the general population, LCI has been linked to non-alcoholic fatty liver disease, insulin resistance, and subclinical atherosclerosis, supporting its utility as a composite atherogenic metric (30, 31). Our study extends these observations to PD, a population with unique metabolic challenges.

The age-specific interaction observed here is also noteworthy. Age-related differences in lipid metabolism, hormonal milieu, and vascular remodeling may modify the impact of dyslipidemia on outcomes. Similar age-dependent modulation has been reported in large population-based cohorts, where lipid-related risk appeared to be more pronounced among younger individuals (32). Furthermore, the non-linearity observed for all-cause but not cardiovascular mortality is compatible with the notion that LCI may capture broader systemic processes beyond atherosclerosis. In PD populations, systemic inflammation and protein-energy malnutrition/low albumin are major determinants of survival, and PD-related infections (notably peritonitis) substantially increase mortality risk; together, these non-atherosclerotic pathways disproportionately shape all-cause mortality (33–35).

From a clinical perspective, these findings highlight the potential of LCI as a simple yet powerful biomarker for integrated risk assessment in PD. Unlike traditional lipid indices, LCI simultaneously reflects dyslipidemia severity, inflammation, and nutritional imbalance. Routine calculation of LCI from standard lipid panels may help clinicians identify high-risk patients who could benefit from targeted interventions, such as optimizing lipid control, reducing glucose exposure from dialysate, and correcting protein-energy wasting. The KDIGO guidelines currently recommend lipid management in CKD but provide limited guidance for dialysis patients (36). Our findings argue for re-evaluation of lipid targets in PD, particularly in younger individuals with persistently high LCI.

Potential interventions may include intensified statin therapy, combination regimens with fibrates or omega-3 fatty acids, and PD prescription modifications, such as glucose-sparing solutions or automated PD, to mitigate hypertriglyceridemia. Moreover, emerging therapies targeting inflammation and lipid oxidation may offer new opportunities to lower atherogenic burden and the risk captured by LCI (37, 38).

However, several limitations must be acknowledged. The observational design precludes causal inference, and residual confounding cannot be fully excluded. Missing data, particularly for cardiovascular history (≈ 48.6%), may have attenuated some associations. The complete-case analysis yielded non-significant results, suggesting limited statistical power or potential selection bias. Moreover, LCI was measured only at baseline; dynamic changes during follow-up could provide greater prognostic information. Lastly, our cohort comprised Chinese PD patients, and external validation in other ethnicities and dialysis modalities is warranted. Future prospective and mechanistic studies should evaluate whether interventions that lower LCI can translate into improved survival and reduced cardiovascular events in PD.

## 5 Conclusion

In this multicenter cohort of incident PD patients, higher baseline LCI was independently associated with increased risks of all-cause and cardiovascular mortality. These findings suggest that LCI integrates lipid, inflammatory, and nutritional abnormalities into a single prognostic index, offering potential value for refined risk stratification beyond conventional lipid parameters. Incorporating LCI into clinical assessment may help identify high-risk patients who could benefit from more intensive lipid-metabolic management and tailored therapeutic strategies. Future prospective studies are warranted to validate these findings and to determine whether interventions targeting LCI can improve outcomes in the PD population.

## Data Availability

The raw data supporting the conclusions of this article will be made available by the authors, without undue reservation.
