# Supplementary material for: Association between lipoprotein combine index and all-cause and cardiovascular mortality in patients undergoing peritoneal dialysis: a multicenter retrospective cohort study
Source: Front Nutr. 2026 Mar 3;13:1768195. doi: 10.3389/fnut.2026.1768195 (PMC12992042; doi:10.3389/fnut.2026.1768195)
Supplement: Supplementary file 1 [file Table_1.docx]

| **Exposure** | **sHR (95% CI)** | **P value** |
| --- | --- | --- |
| **LCI quartiles** |  |  |
| Q2 vs Q1 | 1.36 (0.98 - 1.89) | 0.070 |
| Q3 vs Q1 | 1.11 (0.79 - 1.57) | 0.530 |
| Q4 vs Q1 | 1.54 (1.11 - 2.12) | 0.009 |
| **LCI (continuous)** |  |  |
| Per 1-SD increase in LCI | 1.16 (1.05 - 1.27) | 0.003 |

**Abbreviations:** sHR, subdistribution hazard ratio; CI, confidence interval; LCI, Lipoprotein Combine Index; SD, standard deviation; SBP, systolic blood pressure; DBP, diastolic blood pressure; BMI, body mass index; ALP, alkaline phosphatase; CRP, C-reactive protein; CVD, cardiovascular disease.

**Notes:**

1. model adjusted for age, sex, SBP, DBP, BMI, diabetes, history of CVD, hemoglobin, uric acid, albumin, ALP, CRP, calcium, phosphate, aspirin use, statin use, and residual renal function.
2. Fine-Gray subdistribution hazards models were applied with non-cardiovascular deaths treated as competing events.
3. Results remained consistent with the Cox proportional-hazards models, indicating that higher LCI is independently associated with increased cardiovascular mortality risk even after accounting for competing events.

**Table S1**. Sensitivity analysis using Fine-Gray competing-risks models for cardiovascular mortality in peritoneal dialysis patients
