# Supplementary material for: Association between lipoprotein combine index and all-cause and cardiovascular mortality in patients undergoing peritoneal dialysis: a multicenter retrospective cohort study
Source: Front Nutr. 2026 Mar 3;13:1768195. doi: 10.3389/fnut.2026.1768195 (PMC12992042; doi:10.3389/fnut.2026.1768195)
Supplement: Supplementary file 2 [file Table_2.docx]

| **Variable** | **HR (95% CI)** | ***P* value** |
| --- | --- | --- |
| **All-cause mortality** |  |  |
| LCI Q2 vs Q1 | 1.44 (1.09 - 1.90) | 0.011 |
| LCI Q3 vs Q1 | 1.51 (1.14 - 1.99) | 0.004 |
| LCI Q4 vs Q1 | 1.74 (1.33 - 2.29) | < 0.001 |
| *P* for trend | < 0.001 |  |
| Continuous LCI  (per 1-SD increase) | 1.17 (1.08 - 1.26) | < 0.001 |
| **Cardiovascular mortality** |  |  |
| LCI Q2 vs Q1 | 1.40 (0.96 - 2.05) | 0.078 |
| LCI Q3 vs Q1 | 1.21 (0.82 - 1.79) | 0.325 |
| LCI Q4 vs Q1 | 1.73 (1.20 - 2.49) | 0.003 |
| *P* for trend | 0.009 |  |
| Continuous LCI  (per 1-SD increase) | 1.20 (1.08 - 1.34) | 0.001 |

Table S2. Sensitivity analysis of the association between Lipoprotein Combine Index (LCI) and mortality after excluding patients who died within 6 months of peritoneal dialysis initiation (Model 2, multiple imputation).

**Abbreviations:** HR, hazard ratio; CI, confidence interval; LCI, Lipoprotein Combine Index; SBP, systolic blood pressure; DBP, diastolic blood pressure; BMI, body mass index; ALP, alkaline phosphatase; CRP, C-reactive protein; CVD, cardiovascular disease; RRF, residual renal function.

**Notes:**

1. Cox proportional hazards models were adjusted for age, sex, SBP, DBP, BMI, diabetes, history of CVD, hemoglobin, uric acid, albumin, ALP, calcium, phosphate, CRP, aspirin use, statin use, and residual renal function.
2. Multiple imputation (m = 5) using predictive mean matching was applied for missing covariates.
3. This sensitivity analysis excluded patients who died within 6 months after PD initiation (n = 1,892; 95.3% of total cohort).
