# Supplementary material for: Association between lipoprotein combine index and all-cause and cardiovascular mortality in patients undergoing peritoneal dialysis: a multicenter retrospective cohort study
Source: Front Nutr. 2026 Mar 3;13:1768195. doi: 10.3389/fnut.2026.1768195 (PMC12992042; doi:10.3389/fnut.2026.1768195)
Supplement: Supplementary file 3 [file Table_3.docx]

| **Variable** | **HR (95% CI)** | ***P* value** |
| --- | --- | --- |
| **All-cause mortality** |  |  |
| LCI T2 vs T1 | 1.33 (1.05 - 1.67) | 0.016 |
| LCI T3 vs T1 | 1.55 (1.23 - 1.94) | < 0.001 |
| *P* for trend | < 0.001 |  |
| Continuous LCI  (per 1-SD increase) | 1.16 (1.07 - 1.25) | < 0.001 |
| **Cardiovascular mortality** |  |  |
| LCI T2 vs T1 | 1.19 (0.86 - 1.64) | 0.284 |
| LCI T3 vs T1 | 1.47 (1.08 - 2.01) | 0.015 |
| *P* for trend | 0.014 |  |
| Continuous LCI  (per 1-SD increase) | 1.20 (1.07 - 1.33) | 0.001 |

Table S3. Sensitivity analysis of the association between Lipoprotein Combine Index (LCI) and mortality using tertile-based categorization (Model 2, multiple imputation).

**Abbreviations:** HR, hazard ratio; CI, confidence interval; LCI, Lipoprotein Combine Index; SBP, systolic blood pressure; DBP, diastolic blood pressure; BMI, body mass index; ALP, alkaline phosphatase; CRP, C-reactive protein; CVD, cardiovascular disease; RRF, residual renal function.

**Notes:**

1. Cox proportional hazards models were adjusted for age, sex, SBP, DBP, BMI, diabetes mellitus, history of CVD, hemoglobin, uric acid, serum albumin, ALP, calcium, phosphate, CRP, aspirin use, statin use, and RRF.
2. Multiple imputation (m = 5) using the predictive mean matching method was applied to handle missing covariates.
3. This sensitivity analysis evaluated whether the associations between LCI and both all-cause and cardiovascular mortality were robust to different categorization strategies.
