# Supplementary material for: Association between lipoprotein combine index and all-cause and cardiovascular mortality in patients undergoing peritoneal dialysis: a multicenter retrospective cohort study
Source: Front Nutr. 2026 Mar 3;13:1768195. doi: 10.3389/fnut.2026.1768195 (PMC12992042; doi:10.3389/fnut.2026.1768195)
Supplement: Supplementary file 4 [file Table_4.docx]

| **Variable** | **HR (95% CI)** | ***P* value** |
| --- | --- | --- |
| **All-cause mortality** |  |  |
| LCI Q2 vs Q1 | 1.39 (0.91 - 2.13) | 0.125 |
| LCI Q3 vs Q1 | 1.15 (0.77 - 1.72) | 0.507 |
| LCI Q4 vs Q1 | 1.05 (0.70 - 1.57) | 0.819 |
| *P* for trend | 0.600 |  |
| Continuous LCI  (per 1-SD increase) | 1.02 (0.90 - 1.16) | 0.777 |
| **Cardiovascular mortality** |  |  |
| LCI Q2 vs Q1 | 1.10 (0.63 - 1.92) | 0.737 |
| LCI Q3 vs Q1 | 0.87 (0.51 - 1.48) | 0.612 |
| LCI Q4 vs Q1 | 0.87 (0.52 - 1.46) | 0.594 |
| *P* for trend | 0.393 |  |
| Continuous LCI  (per 1-SD increase) | 1.00 (0.84 - 1.19) | 0.970 |

Table S4: Association between Lipoprotein Combine Index (LCI) and mortality outcomes in peritoneal dialysis patients (complete-case analysis).

**Abbreviations:** HR, hazard ratio; CI, confidence interval; LCI, Lipoprotein Combine Index; SBP, systolic blood pressure; DBP, diastolic blood pressure; BMI, body mass index; ALP, alkaline phosphatase; CRP, C-reactive protein; CVD, cardiovascular disease; RRF, residual renal function.

**Notes:**

1. Complete-case analysis included patients with no missing data for any covariates used in Model 2 of the primary analysis (n = 550, 27.7 % of the total cohort).
2. LCI was categorized into quartiles (Q1-Q4) based on its distribution in the overall study population; Q1 served as the reference group.
3. Hazard ratios and 95 % confidence intervals were estimated using Cox proportional hazards models to assess the association between LCI and mortality outcomes.
