# Supplementary material for: Association between lipoprotein combine index and all-cause and cardiovascular mortality in patients undergoing peritoneal dialysis: a multicenter retrospective cohort study
Source: Front Nutr. 2026 Mar 3;13:1768195. doi: 10.3389/fnut.2026.1768195 (PMC12992042; doi:10.3389/fnut.2026.1768195)
Supplement: Supplementary file 5 [file Table_5.docx]

Table S5: Association between Lipoprotein Combine Index and mortality outcomes in peritoneal dialysis patients under the MNAR hypothesis.

| **Variable** | **HR (95% CI)** | ***P* value** |
| --- | --- | --- |
| **All-cause mortality** |  |  |
| LCI Q2 vs Q1 | 1.27 (0.99 - 1.63) | 0.057 |
| LCI Q3 vs Q1 | 1.36 (1.06 - 1.74) | 0.014 |
| LCI Q4 vs Q1 | 1.42 (1.13 - 1.80) | 0.004 |
| **Cardiovascular mortality** |  |  |
| LCI Q2 vs Q1 | 1.31 (0.93 - 1.84) | 0.121 |
| LCI Q3 vs Q1 | 1.09 (0.77 - 1.56) | 0.617 |
| LCI Q4 vs Q1 | 1.38 (1.02 – 1.93) | 0.048 |

**Abbreviations**: HR, hazard ratio; CI, confidence interval; LCI, Lipoprotein Combine Index; SD, standard deviation; SBP, systolic blood pressure; DBP, diastolic blood pressure; BMI, body mass index; ALP, alkaline phosphatase; CRP, C-reactive protein; CVD, cardiovascular disease.

**Notes:**

Model adjusted for age, sex, SBP, DBP, BMI, diabetes, history of CVD, hemoglobin, uric acid, albumin, ALP, CRP, calcium, phosphate, aspirin use, statin use, and residual renal function.

Hazard ratios were derived from center-stratified Cox proportional hazards models using multiple imputation (m = 5) for missing covariates under the MNAR hypothesis. HRs and 95% CIs were pooled using Rubin’s rules.
